# University of Maryland, Dental Department

**Published:** 1897-04

**Authors:** 


### Editorial, Etc.

### UNIVERSITY OF MARYLAND, DENTAL DEPARTMENT.

The annual commencement of the Department of Dental Surgery of the University of Maryland was held in the Lyceum Theatre, March 31st, 1897, at 8 p. m., in the presence of an audience that filled every available space of the house. The occasion was marked by an unusually large floral display, there being no former commencement held in Baltimore where as many fragrant flowers were sent by the friends and admirers of the graduates. The entire front of the stage was basked with roses, carnations, annunciation lilies, violets and other exotics, which were subsequently presented to the individuals for whom they were intended.

When the curtain went up the students were seated in a semicircle around the stage, attired in the conventional graduation cap and gown. Prayer was offered by the Rev. Samuel A. Wilson, and the mandamus was read by Prof. Ferdinand J. S. Gorgas, dean of the faculty.

In the absence of Bernard Carter, provost of the university, the degrees were conferred by Hon. John P. Poe, acting provost.

The address to the graduates was made by the Rev. M. D. Wharton, D D., who took for his subject "Voices From a Buried City." After a few preliminary remarks about "plugging" and other suggestions of the dental profession, Dr. Wharton branched off upon Pompeii and spoke of the echoes of the ancient city, which he visited in his recent travels. His address was filled with anecdotes and poetical quotations, including an original version of Thomas Moore's lines to suit the circumstances : "You may break, you may ruin yout teeth if you will; but the dread of the dentist will hang round you still."

Prizes were awarded by Prof. Francis T. Miles, as follows :

Seniors—Two University Gold Medals for highest grade, M. N. King and D. E. McConnell. Honorable mention for second highest grade, F. R. Thomas. S. S. White Prize, F. B. Weller. Snowden & Cowman Manufacturing Company Prize, F. S. Anderson. Prof. Harris' Prize, P. Russell. Prof. Gorgas' Prize, J. W. Faucette. Dr. C. J. Grieves' Prize for Bridge Work, R. L. Henry. Dr. C. J, Grieves' Prize for Senior Crown Work, W, G. Boyd.

Junior Prizes—Dr. J. C. Uhler's Prize, C. D. Holmes. Dr. J. H. Davis' Prize, C. W. Leonard. H. W. Cassel Prize, E. R. Williams. C. R. Deeley Prize, C. E. Fording. Dental Department Prize, F. R. Harkinson.

Freshmen Prizes—S. S. White Prize, T. Takashima. Dental Department Prize, J. H. Baker.

Honorable Mention—F. R. Thomas, D. E. McConnell, S. B. Wrightson, F. S. Robinson, R. L. Henry, A. A. Williams, Jr., R. R. Reiff, E. A. Charbonnel, R. G. Norfleet, C. L. Hartman, E. J. Applewhite, H. I. O'Connor, J. L. Duff, R. M. Muir, W. C. Seaton, F. W. Quinlan, J. B. Grom, P. A. Michel, R. T. Gallagher, G. A. Sprinkel, Jr., M. B. Milner, R. F. Moore, C. W. Leonard, F. E. Smith, R. Morris, W. T. Lineweaver.

The class oration was delivered by H. C. Raoul Breault, of Connecticut, and was an excellent address.

During the evening a musical selection was rendered, under the direction of Prof. William F. Thiede's Orchestra.

The class officers for 1896-7 are : President, R. Lee Henry, Georgia; vice-president, Henry I. O'Connor, Georgia; secretary, Samuel B. Wrightson, Maryland; treasurer, Edgar J. Applewhite, Virginia; executive committee, Walter G. Boyd, Maryland, chairman; Fred. R, Thomas, Nova Scotia; Robert G. Norfleet, Virginia;

Newton F. Foote, New York; John McC. Foreman, Pennsylvania; Patrick J. Vaughan, Georgia.

The graduates are: Albert A. Aiken, Texas; Fred. S. Anderson, New Brunswick; Edgar Jarratt Applewhite, Virginia; Charles E. Ayres, Pennsylvania; Albert D. Baker, Maryland; J. Henry Besore, Maryland; Samuel D. Bodine, Pennsylvania; Walter G. Boyd, Maryland; H. C. Raoul Breault, Connecticut; John H. Bushong, Virginia; E. Adolph Charbonnel, Washington; Clarence E. Collins, Maryland; James L. Duff, Canada; Arthur Paul Farley, Connecticut; John William Faucette, North Carolina; Eugene Finnegan, New York; Newton Fernando Foote, New York; John McClosky Foreman, Pennsylvania; A. Hutchings Frith, Bermuda; John Gardner, Maryland; Arthur Perry Gordy, Georgia; W. Gordon Gould, Connecticut: Curtis LeRoy Hartman, Pennsylvania; Charles F. Heisler, Pennsylvania; Robert Lee Henry, Georgia; John Griffin Hoffman, Virginia; Marion N. King, Virginia; John Moubray MacDonald, New Zealand; Daniel T., Maloney, Connecticut; Chesley Martin, Virginia; Daniel Edward McConnell, South Carolina; C. Howard McNutt, Nova Scotia; Robert W. Morton, Pennsylvania; Wirt H. Moseley, Virginia; Abraham V. Mowel, New York; R. Murray Muir, Canada; Robert Cassell Nicodemus, Maryland; Robert Gordon Norfleet Virginia; Samuel P. Norris, North Carolina; Henry Ignatius O'Connor, Georgia; Henry Newton Pheneger, Pennsylvania; Thomas Lloyd Posey, Kentucky; R. Russell Reiff Pennsylvania; James Leftwich Rogers, Tennessee; Frank Sidney Robinson, Florida; Louis H. Russell, South Carolina; Percy Russell, Maryland; William Close Seaton, Jr., New York; Walter Owens Stack, Delaware; J. Russell Steele, Pennsylvania; Stanley Steele, Maryland; Max Stein, Russia; Charles C. Switzer, New York; Elisha S. Taylor, Maryland; Fred. Rudolf Thomas, Nova Scotia; Alexander Tribble, Missouri; Patrick, J. Vaughan, Georgia; J. Somes Watts, Canada; Julius Weinberger, New York; Franklin Babbitt Weller, New York; Alpha Ayer Williams, Jr., Georgia; and Samuel B. Wrightson, Maryland.

